# Supplementary material for: Metagenomic Analyses Reveal the Influence of Depth Layers on Marine Biodiversity on Tropical and Subtropical Regions
Source: Microorganisms. 2023 Jun 27;11(7):1668. doi: 10.3390/microorganisms11071668 (PMC10386303; doi:10.3390/microorganisms11071668)
Supplement: Supplementary file 1 [file microorganisms-11-01668-s001.zip › SupFigures.pdf]

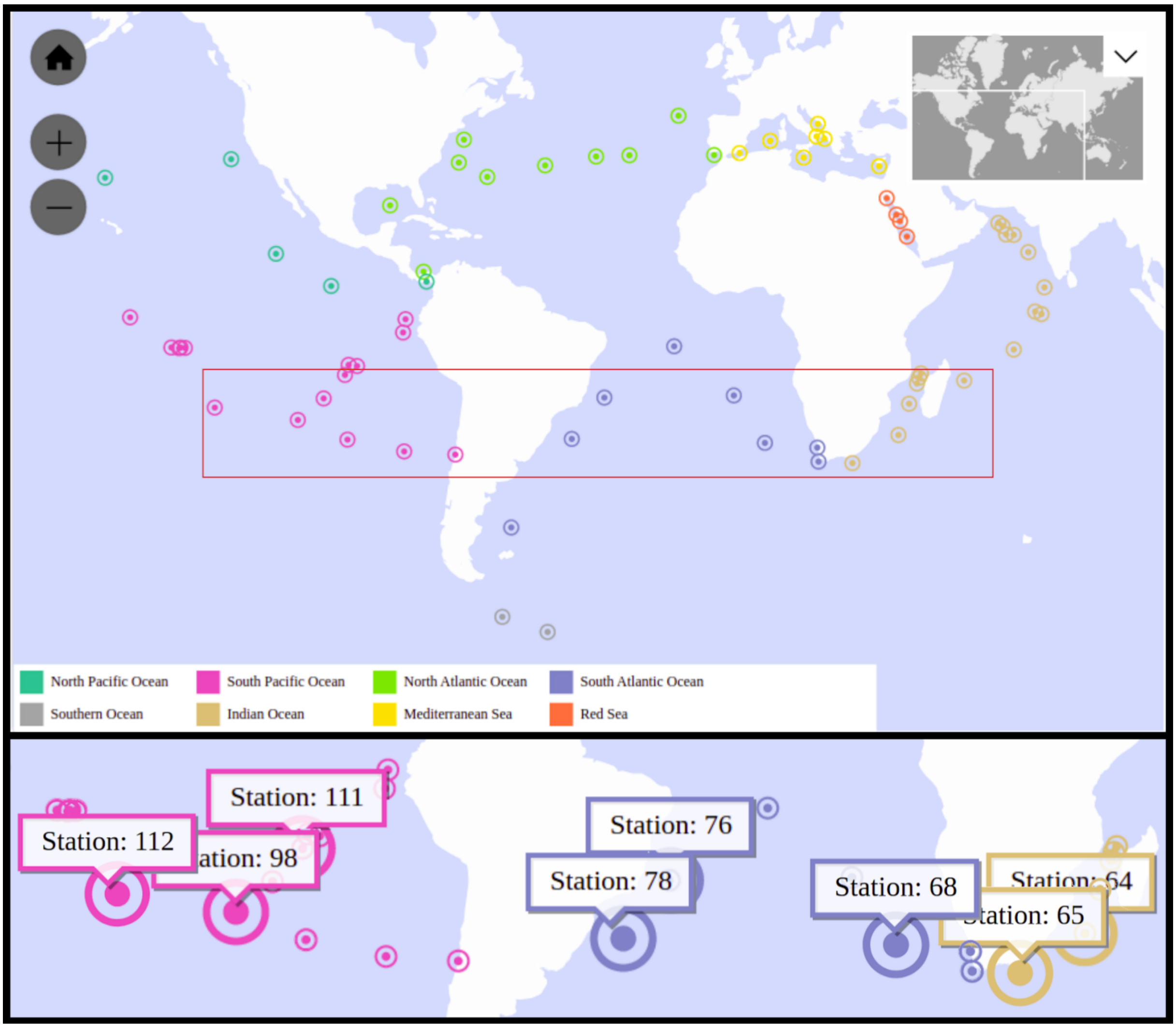

Figure S1. Latitude filter of collection stations. **A)** Filter 1: selection of stations within an interval of 5° positive or negative in relation to stations 76 and 78. **B)** Filter 2: from filter 1, selection of stations with samples in the three depth layers simultaneously.

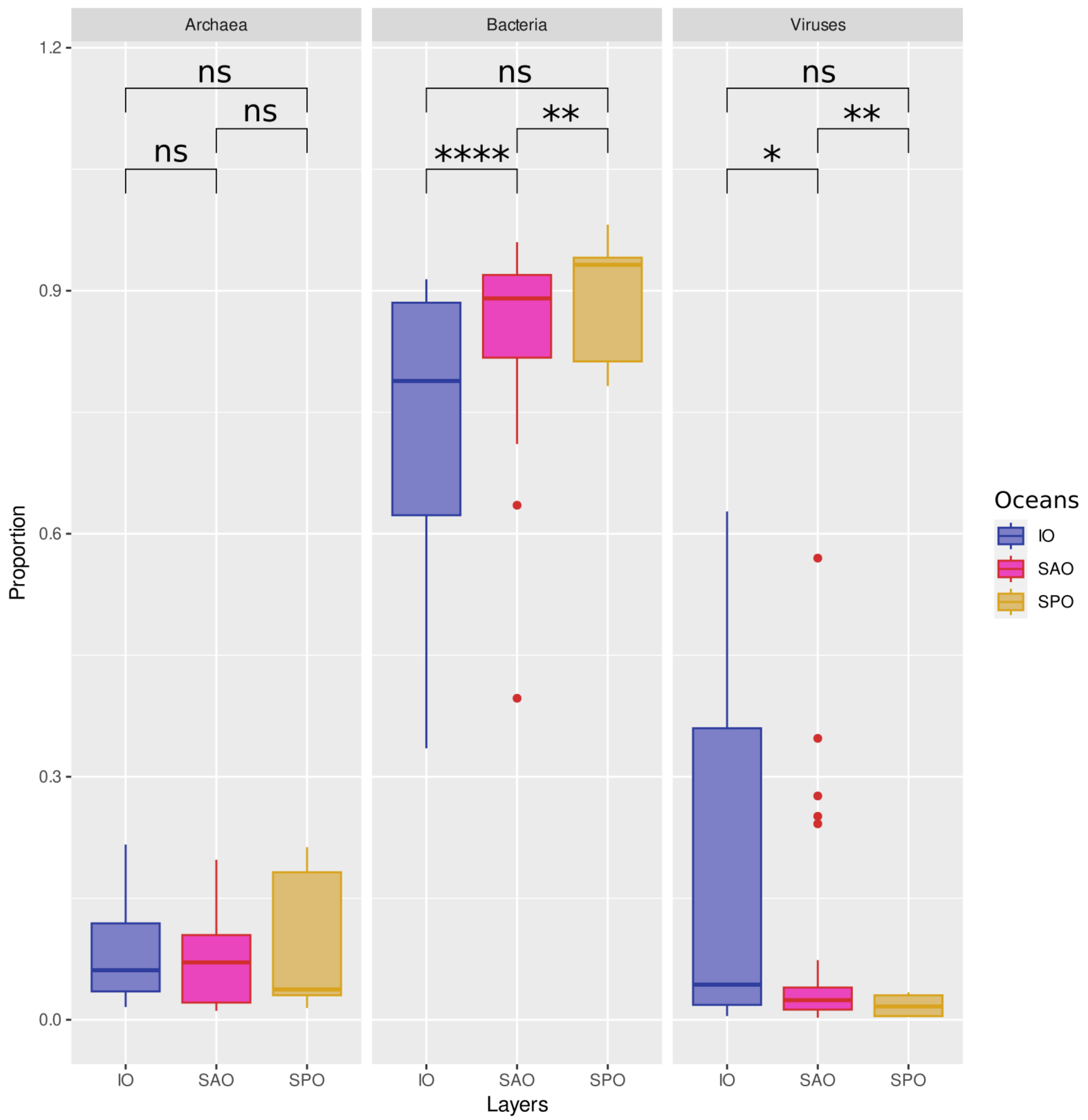

Figure S2. Distribution of domain proportions considering the samples of each ocean. The oceans are represented by the acronyms: (IO - Indian Ocean, SAO - South Atlantic Ocean, and SPO - South Pacific Ocean). Statistical significance is represented as follows. ns (adjusted p-value > 0.05); * (adjusted p-value <= 0.05); ** (adjusted p-value <= 0.01); *** (adjusted p-value <= 0.001); **** (adjusted p-value <= 0.0001).

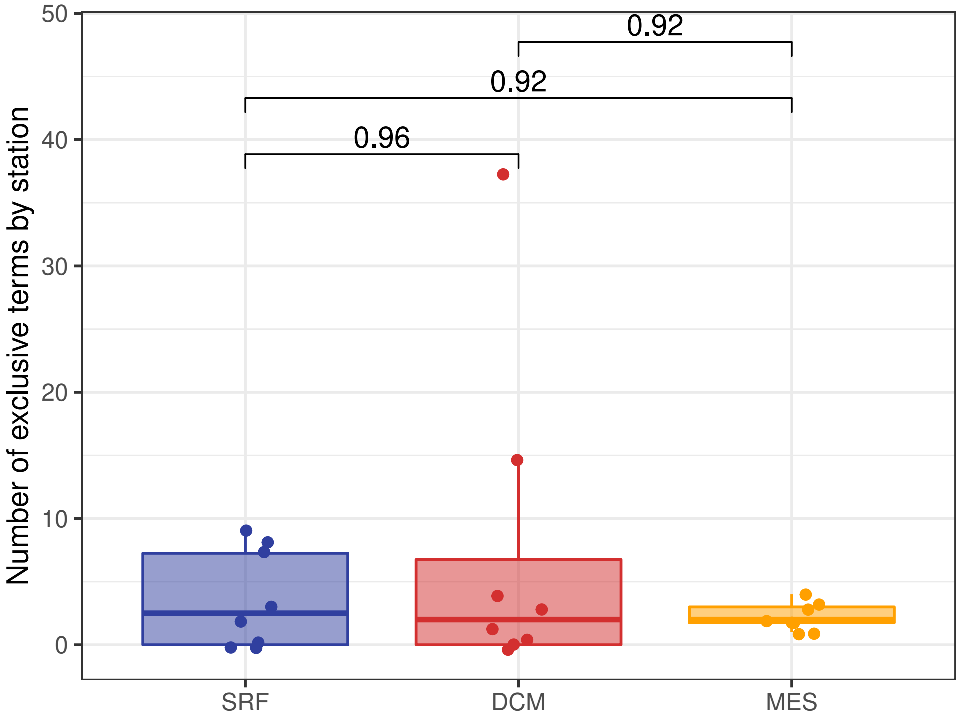

Figure S3: Distribution of exclusive terms by stations in each depth layer. Exclusive terms are the ones that appear only in a given depth layer of a given station. Comparisons are performed with the Wilcoxon rank sum test and Bonferroni-adjusted p-values are represented in each comparison. No significant comparison was observed.
